# Supplementary material for: Tachycardia in hyperthyroidism: Not so common
Source: PLoS One. 2022 Sep 2;17(9):e0273724. doi: 10.1371/journal.pone.0273724 (PMC9439220; doi:10.1371/journal.pone.0273724)
Supplement: Attachment — Submitted filename: Response to Reviewers 8-11-22.docx [file pone.0273724.s002.docx]

| **Date:** | Aug 02 2022 11:03AM |
| --- | --- |
| **To:** | "Leon Fogelfeld" lfogelfeld@cookcountyhhs.org |
| **From:** | "PLOS ONE" plosone@plos.org |
| **Subject:** | PLOS ONE Decision: Revision required [PONE-D-22-07597] REBUTTAL -RESPONSE TO REVIEWERS |

***RE: REBUTTAL-RESPONSE TO REVIEWERS***

*Please see our rebuttal in italics for every reviewer point indicating also the exact locations of the revisions.*

PONE-D-22-07597

Tachycardia in Hyperthyroidism: Not So Common

PLOS ONE

Dear Dr. Leon Fogelfeld,

Thank you for submitting your manuscript to PLOS ONE. After careful consideration, we feel that it has merit but does not fully meet PLOS ONE’s publication criteria as it currently stands. Therefore, we invite you to submit a revised version of the manuscript that addresses the points raised during the review process.

==============================

- Indicate the novelty of the manuscript clearly.
- *The novelty of the manuscript is added in the abstract objective and in the conclusion (last paragraph).*
- Tables should be in accordance with author guidelines.
- *Was done.*
- Update the references in accordance with the format and include the latest references.
- *Was done.*

==============================

Please submit your revised manuscript by August 15, 2022. If you will need more time than this to complete your revisions, please reply to this message or contact the journal office at [plosone@plos.org](mailto:plosone@plos.org). When you're ready to submit your revision, log on to <https://www.editorialmanager.com/pone/> and select the 'Submissions Needing Revision' folder to locate your manuscript file.

Please include the following items when submitting your revised manuscript:

- A rebuttal letter that responds to each point raised by the academic editor and reviewer(s). You should upload this letter as a separate file labeled 'Response to Reviewers'.
- *Was done*
- A marked-up copy of your manuscript that highlights changes made to the original version. You should upload this as a separate file labeled 'Revised Manuscript with Track Changes'.
- *Was done*
- An unmarked version of your revised paper without tracked changes. You should upload this as a separate file labeled 'Manuscript'.
- *Was done*

If you would like to make changes to your financial disclosure, please include your updated statement in your cover letter. Guidelines for resubmitting your figure files are available below the reviewer comments at the end of this letter.

If applicable, we recommend that you deposit your laboratory protocols in protocols.io to enhance the reproducibility of your results. Protocols.io assigns your protocol its own identifier (DOI) so that it can be cited independently in the future. For instructions see: [https://journals.plos.org/plosone/s/submission-guidelines#loc-laboratory-protocols](https://journals.plos.org/plosone/s/submission-guidelines). Additionally, PLOS ONE offers an option for publishing peer-reviewed Lab Protocol articles, which describe protocols hosted on protocols.io. Read more information on sharing protocols at [https://plos.org/protocols?utm_medium=editorial-email HYPERLINK "https://plos.org/protocols?utm_medium=editorial-email&utm_source=authorletters&utm_campaign=protocols"& HYPERLINK "https://plos.org/protocols?utm_medium=editorial-email&utm_source=authorletters&utm_campaign=protocols"utm_source=authorletters HYPERLINK "https://plos.org/protocols?utm_medium=editorial-email&utm_source=authorletters&utm_campaign=protocols"& HYPERLINK "https://plos.org/protocols?utm_medium=editorial-email&utm_source=authorletters&utm_campaign=protocols"utm_campaign=protocols](https://plos.org/protocols?utm_medium=editorial-email&utm_source=authorletters&utm_campaign=protocols).

We look forward to receiving your revised manuscript.

Kind regards,

Dr. Anu Sayal, Ph.D.
Academic Editor
PLOS ONE

Journal requirements:

When submitting your revision, we need you to address these additional requirements.

1. Please ensure that your manuscript meets PLOS ONE's style requirements, including those for file naming. The PLOS ONE style templates can be found at

<https://journals.plos.org/plosone/s/file?id=wjVg/PLOSOne_formatting_sample_main_body.pdf> and

<https://journals.plos.org/plosone/s/file?id=ba62/PLOSOne_formatting_sample_title_authors_affiliations.pdf>"

*Manuscript reviewed and appears to meet the guidelines.*

2. Please provide additional details regarding participant consent. In the ethics statement in the Methods and online submission information, please ensure that you have specified (1) whether consent was informed and (2) what type you obtained (for instance, written or verbal, and if verbal, how it was documented and witnessed). If your study included minors, state whether you obtained consent from parents or guardians. If the need for consent was waived by the ethics committee, please include this information.

If you are reporting a retrospective study of medical records or archived samples, please ensure that you have discussed whether all data were fully anonymized before you accessed them and/or whether the IRB or ethics committee waived the requirement for informed consent. If patients provided informed written consent to have data from their medical records used in research, please include this information.

*More information about IRB approval and consent waiver provided on page 4 at the end of the Study Design paragraph.*

3. We note that you have stated that you will provide repository information for your data at acceptance. Should your manuscript be accepted for publication, we will hold it until you provide the relevant accession numbers or DOIs necessary to access your data. If you wish to make changes to your Data Availability statement, please describe these changes in your cover letter and we will update your Data Availability statement to reflect the information you provide.

*De-identified data file will be included in supplemental materials.*

Please review your reference list to ensure that it is complete and correct. If you have cited papers that have been retracted, please include the rationale for doing so in the manuscript text, or remove these references and replace them with relevant current references. Any changes to the reference list should be mentioned in the rebuttal letter that accompanies your revised manuscript. If you need to cite a retracted article, indicate the article’s retracted status in the References list and also include a citation and full reference for the retraction notice.

*Reference list reviewed and revised in format where need to match Vancouver style. No references were deleted or added.*

Reviewers' comments:

Reviewer's Responses to Questions

**Comments to the Author**

1. Is the manuscript technically sound, and do the data support the conclusions?

The manuscript must describe a technically sound piece of scientific research with data that supports the conclusions. Experiments must have been conducted rigorously, with appropriate controls, replication, and sample sizes. The conclusions must be drawn appropriately based on the data presented.

Reviewer #1: Yes

Reviewer #2: Yes

2. Has the statistical analysis been performed appropriately and rigorously?

Reviewer #1: Yes

Reviewer #2: I Don't Know

3. Have the authors made all data underlying the findings in their manuscript fully available?

The [PLOS Data policy](http://www.plosone.org/static/policies.action) requires authors to make all data underlying the findings described in their manuscript fully available without restriction, with rare exception (please refer to the Data Availability Statement in the manuscript PDF file). The data should be provided as part of the manuscript or its supporting information, or deposited to a public repository. For example, in addition to summary statistics, the data points behind means, medians and variance measures should be available. If there are restrictions on publicly sharing data—e.g. participant privacy or use of data from a third party—those must be specified.

Reviewer #1: Yes

Reviewer #2: Yes

4. Is the manuscript presented in an intelligible fashion and written in standard English?

PLOS ONE does not copyedit accepted manuscripts, so the language in submitted articles must be clear, correct, and unambiguous. Any typographical or grammatical errors should be corrected at revision, so please note any specific errors here.

Reviewer #1: Yes

Reviewer #2: Yes

5. Review Comments to the Author

Please use the space provided to explain your answers to the questions above. You may also include additional comments for the author, including concerns about dual publication, research ethics, or publication ethics. (Please upload your review as an attachment if it exceeds 20,000 characters)

Reviewer #1: Please mention the full form of TSH and FT4 in the abstract for the first time.

*Corrected.*

Please separate methods from findings in the abstract.

*Corrected.*

Please address the novelty of your study.

*The novelty has been added to the abstract objective and to the end of the discussion in the last conclusion paragraph.*

Please mention that you reported categorical variables by frequency and percent.

*Added to Statistical Analysis, page 7.*

Please revise the tables and justify them based on the authors' guidelines.

*On review, the tables appear to meet the table formatting guidelines. Please advise if anything requires revision.*

Please update your references. You have references from 1946.

*The reference from 1946 is appropriate in the context (Discussion, page 10) of the discussion of past historical findings concerning tachycardia in those with hyperthyroidism.*

You have to revise some of your references based on the Vancouver style.

*References have been revised.*

With regards,

Reviewer #2: An interesting report in issuing current practice that may effect the previous usual findings. It will be good to have more extensive parameters being reports in this report.

*The study reported the outcomes for all the relevant parameters pertaining to hyperthyroidism and tachycardia available for our study group.*

The usage of the abbreviation can be reduced to enhanced easy to understand reading among readers.

*Unsure which abbreviation is lacking clarity. Abbreviations after the tables and figures have been removed since it does not appear to fit the format of PLOS One.*

6. PLOS authors have the option to publish the peer review history of their article ([what does this mean?](https://journals.plos.org/plosone/s/editorial-and-peer-review-process)). If published, this will include your full peer review and any attached files.


If you choose “no”, your identity will remain anonymous but your review may still be made public.


**Do you want your identity to be public for this peer review?** For information about this choice, including consent withdrawal, please see our [Privacy Policy](https://www.plos.org/privacy-policy).

Reviewer #1: **Yes:** Afagh Hassanzadeh Rad

Reviewer #2: No

[NOTE: If reviewer comments were submitted as an attachment file, they will be attached to this email and accessible via the submission site. Please log into your account, locate the manuscript record, and check for the action link "View Attachments". If this link does not appear, there are no attachment files.]

While revising your submission, please upload your figure files to the Preflight Analysis and Conversion Engine (PACE) digital diagnostic tool, <https://pacev2.apexcovantage.com/>. PACE helps ensure that figures meet PLOS requirements. To use PACE, you must first register as a user. Registration is free. Then, login and navigate to the UPLOAD tab, where you will find detailed instructions on how to use the tool. If you encounter any issues or have any questions when using PACE, please email PLOS at [figures@plos.org](mailto:figures@plos.org). Please note that Supporting Information files do not need this step.

*Figures checked via PACE with PACE figures uploaded.*

*In compliance with data protection regulations, you may request that we remove your personal registration details at any time.* [*(Remove my information/details)*](https://www.editorialmanager.com/pone/login.asp?a=r)*. Please contact the publication office if you have any questions.*
